# A Breathtaking Lift: Sex and Body Mass Index Differences in Cardiopulmonary Response in a Large Cohort of Unselected Subjects with Acute Exposure to High Altitude

**DOI:** 10.1089/ham.2021.0039

**Published:** 2021-12-13

**Authors:** Carlo Vignati, Massimo Mapelli, Benedetta Nusca, Alice Bonomi, Elisabetta Salvioni, Irene Mattavelli, Susanna Sciomer, Andrea Faini, Gianfranco Parati, Piergiuseppe Agostoni

**Affiliations:** ^1^ Centro Cardiologico Monzino, IRCCS, Milan, Italy.; ^2^ Department of Clinical Sciences and Community Health, Cardiovascular Section, University of Milan, Milan, Italy.; ^3^ Dipartimento di Scienze Cardiovascolari, Respiratorie, Nefrologiche, Anestesiologiche e Geriatriche, “Sapienza” Rome University, Rome, Italy.; ^4^ Istituto Auxologico Italiano, IRCCS, Department of Cardiovascular, Neural and Metabolic Sciences, San Luca Hospital, Milan, Italy.; ^5^ Department of Medicine and Surgery, University of Milano-Bicocca, Milan, Italy.

**Keywords:** acute hypoxia, BMI, high altitude, sex differences

## Abstract

Vignati, Carlo, Massimo Mapelli, Benedetta Nusca, Alice Bonomi, Elisabetta Salvioni, Irene Mattavelli, Susanna Sciomer, Andrea Faini, Gianfranco Parati, and Piergiuseppe Agostoni. A breathtaking lift: sex and body mass index differences in cardiopulmonary response in a large cohort of unselected subjects with acute exposure to high altitude. *High Alt Med Biol*. 22:379–385, 2021.

***Background:*** Every year, thousands of people travel to high altitude and experience hypoxemia. At high altitude, the partial pressure of oxygen decreases.

The aim of this observational study was to determine if there is a relationship between anthropometric features and basic cardiorespiratory variables, including oxygen saturation (SpO_2_), heart rate (HR), and blood pressure (BP), following acute exposure to high altitude.

***Materials and Methods:*** At the 3,466 m top of a cableway station, we installed an automated system for measuring peripheral SpO_2_, HR, BP, height, weight, and body mass index (BMI).

***Results:*** Between January and October 2020, out of 4,874 volunteers (age 39.9 ± 15.4 years, male 54.4%), 3,267 provided complete data (1,808 cases during winter and 1,459 during summer). SpO_2_ was 86.8% ± 6.8%. At multivariable analysis, SpO_2_ was significantly associated with age, sex, season, BMI, and HR but not with BP. We identified 391 (12%) subjects with SpO_2_ ≤80%: they were older, with a higher BMI and HR but without sex or BP differences. Finally, winter season was associated with greater frequency of SpO_2_ ≤80% (13.3% vs. 10.3%, *p* = 0.008).

***Conclusion:*** Our data show that high BMI, older age, and male sex were associated with greater degrees of hypoxemia following exposure to high altitude, particularly during the winter.

## Introduction

Among the currently available traveling options, modern cable cars allow an increasingly large number of individuals, including sedentary people, elderly subjects, and cardiorespiratory patients, to easily and rapidly reach high-altitude locations (Agostoni et al., 2010). Indeed, the possibility of a rapid ascent to high altitudes is becoming increasingly common and easy. Every year, thousands of people approach high altitude for different reasons, including work, sport, and tourism (Parati et al., 2018). When moving from sea level to high altitude, reductions in atmospheric pressure (and thus in the partial pressure of oxygen), in air humidity, and in ambient temperature are known to occur (Morgan et al., 1990; Higgins et al., 2010). However, besides the effects of high altitude, barometric pressure decreases with lower temperature, bad weather, and during the winter season. As a matter of fact, the effect of these variables becomes physiologically relevant above ∼2,800 m (Richalet et al., 2012; Roach et al., 2017).

The physiological mechanisms of acclimatization to acute high-altitude exposure impose an increased workload on the cardiovascular system, but few data are available in large nonselected populations. Specifically, as yet, there has been no large-scale epidemiological investigation of the cardiopulmonary response upon rapid ascent to high altitude, in terms of systolic blood pressure (SBP), diastolic blood pressure (DBP), heart rate (HR), and peripheral oxygen saturation (SpO_2_). Accordingly, in the present observational study, we evaluated the cardiopulmonary response to acute high-altitude exposure at Punta Helbronner (3,466 m above sea level), a location on Mont Blanc that is readily accessible by a 20-minute cableway ride from Courmayeur (Entreves station, 1,300 m, Skyway Monte Bianco).

## Materials and Methods

In December 2019, a biometric multiparametric recording system (Keito K9; Keito, Barcelona, Spain) was installed at Punta Helbronner. Keito K9 is an automatic multiparametric recoding system for measuring SpO_2_, HR (pulse oximeter), blood pressure (BP; wrist pressure cuff, automatic), height (laser height meter), weight (scale platform), and body mass index (BMI). Keito K9 was installed inside the room temperature Punta Helbronner cableway station. Once initiated by the subject, the automated Keito K9 system provides a sequence of vocal and animated directions to guide subjects through the measurements (the subject may elect to abstain from some of the measurements). Upon completion, the system prints a summary receipt for the subject, and the measurements are transmitted through a Wi-Fi network and collected in an Excel sheet.

**FIG. 1.**
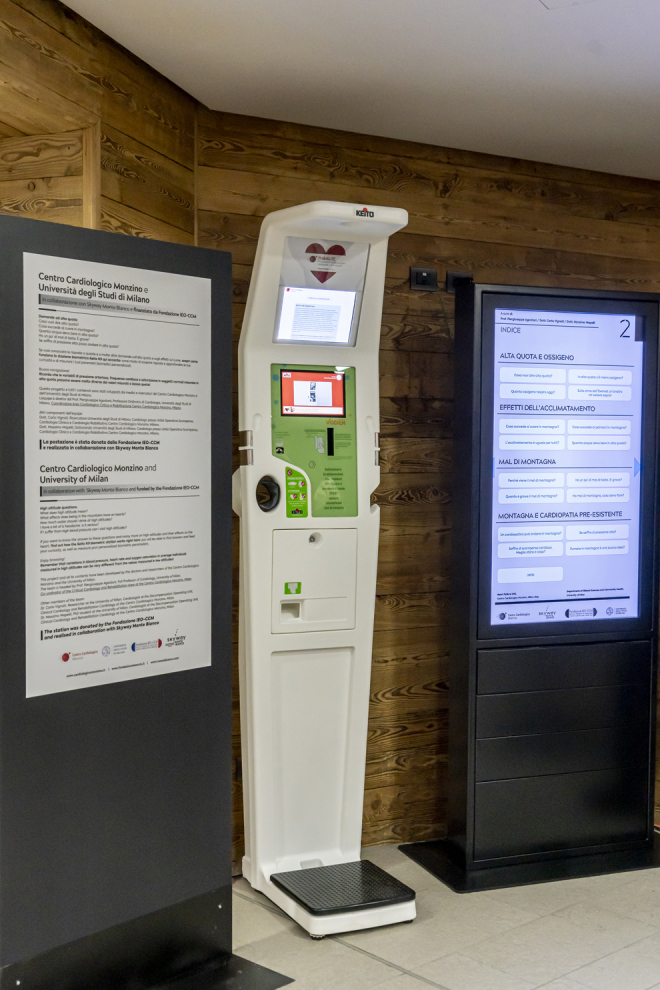
Keito K9 biometric multiparametric recording system (Keito, Barcelona, Spain) installed at Punta Helbronner. Explanatory posters and an interactive screen are placed on both sides of the instrument. Courtesy of Skyway Monte Bianco and Mr. Bazzana Aiace.

The subjects were introduced to the purpose of the study and the Keito measurement system by posters at the Entreves base of the cableway circuit. Data were collected shortly upon arrival at Punta Helbronner station (Fig. 1).

Keito K9 is a class IIa device with CE certification and Food and Drug Administration (FDA) approval. The accuracy of measurements was checked twice a month; specifically, the accuracy of the oximeter was tested versus Pulse CO-Oximeter Radical-7 (Masimo Corporation 52 Discovery, Irvine, CA), whereas BP was calibrated with the Keito factory instrument.

We aimed at investigating whether high-altitude SpO_2_ values correlate with specific anthropometric or cardiorespiratory features of the exposed individuals. The study was approved by the Ethics Committee of Centro Cardiologico Monzino (R1167/20-CCM 1229). Participation was entirely voluntary, and the spontaneous input of data into the recording system was considered as indicating individuals' informed consent to have the stored data analyzed for scientific purposes. Data were collected anonymously, so that participating individuals cannot be identified.

### Statistical analyses

Data are reported as average values ± standard deviation, unless otherwise specified. We used IBM SPSS statistics for all analyses, with significance level set at *p* < 0.05 unless otherwise stated. Mean values were compared using the Student's *t*-test. The Chi-squared test was used to assess frequencies. Correlations were performed using Spearman's rank correlation for parametric data.

## Results

A total of 4,874 consecutive subjects volunteered for the present data collection between January and October 2020 (age 39.9 ± 15.4 years, males 54.4%). Of the volunteers, 3,267 undertook all the requested measurements, and these subjects were used for the data analysis. Subjects' age, weight, height, BMI, SpO_2_, HR, SBP, and DBP are reported in Table 1. One thousand seven hundred ninety-three subjects were males and 1,474 were females. Data from 1,808 individuals were collected during the winter season (between January 1 and March 8) and data from 1,459 individuals during the summer season (between July 6 and October 18). Table 1 also shows age, weight, height, BMI, SpO_2_, HR, SBP, and DBP according to sex and season. As regards SpO_2_, the highest values were observed in females and during the summer season.

**Table 1.** Antropometric Data and Blood Pressure of Subjects at Punta Helbronner

	Total population,* n* = 3,267		Male,* n* = 1,793		Female,* n* = 1,474			Winter,* n* = 1,808		Summer,* n* = 1,459		
	Mean	SD	Mean	SD	Mean	SD	*p*	Mean	SD	Mean	SD	*p*
Age (years)	40.1	15.0	40.2	15.3	40.1	14.7	0.743	38.5	14.5	42.2	15.5	<0.001
Weight (kg)	73.3	15.5	81.4	13.9	63.5	10.9	<0.001	73.3	15.5	73.4	15.4	0.815
Height (cm)	173	10	178	9	166	7	<0.001	173	10	172	10	0.161
BMI (kg/m^2^)	24.5	4.0	25.5	3.8	23.2	3.8	<0.001	24.4	3.9	24.6	4.1	0.118
SpO_2_ (%)	86.8	6.8	86.3	6.6	87.3	7.0	<0.001	86.5	6.9	87.1	6.6	0.015
Heart rate (bpm)	86	6	85	16	87	16	<0.001	88	16	84	15	<0.001
Systolic BP (mmHg)	125	18	125	18	124	18	0.162	124	18	125	19	0.132
Diastolic BP (mmHg)	73	7	75	7	70	7	<0.001	73	7	73	7	0.846

BMI, body mass index; BP, blood pressure; SD, standard deviation; SpO_2_, oxygen saturation.

Of note, the winter season cableway activity was interrupted on March 9 due to national government urgent directives related to the COVID-19 pandemic. Finally, the summer season occurred during the COVID-19 pandemic, so that all subjects were requested to wear a facial mask, but this was not the case during the previous winter season.

On univariate analysis, SpO_2_ was associated with age, sex, season, BMI, HR, and DPB, but not with SBP (Table 2). Of note, at multivariable analysis, all variables but DBP remained associated with SpO_2_ (Table 2). Age, sex, BMI, and HR were equally associated with SpO_2_ as evidenced by standardized β values ranging between −0.49 and −0.43 (Table 2).

**Table 2.** Uni and Multivariable Predictors of Oxygen Saturation at Punta Helbronner

	Univariate analysis			Multivariate analysis		
	Beta	Standard error	*p*	Beta	Standard error	*p*
Age (years)	−0.037	0.008	<0.001	−0.495	0.131	<0.001
Season	0.580	0.239	0.015	0.298	0.120	0.013
Gender	−1.016	0.238	<0.001	−0.449	0.129	<0.001
BMI (kg/m^2^)	−0.174	0.030	<0.001	−0.447	0.145	0.002
Heart rate (bpm)	−0.025	0.007	<0.001	−0.432	0.120	<0.001
BP systolic (mmHg)	0.001	0.006	0.830	—	—	—
BP diastolic (mmHg)	−0.049	0.016	0.003	0.149	0.139	0.2812

Average SpO_2_ was 86.8% ± 6.8%. We identified 391 (12%) subjects with SpO_2_ ≤80% (171 out of 1,474 females, 11.6%, and 220 out of 1,793 males, 12.3%, *p* = NS). These subjects were older, and they had higher BMI and HR, but there were no sex or BP differences compared with subjects with higher SpO_2_ values (Table 3). Finally, winter season was associated with greater frequency of SpO_2_ ≤80% (13.3% of subjects in winter vs. 10.3% in summer; *p* = 0.008).

**Table 3.** Population Characteristics According to Oxygen Saturation Values

	SpO_2_* ≤ *80%,* n* = 391		SpO_2_ > 80%,* n* = 2,876		
	Mean	SD	Mean	SD	*p*
Males, *n* (%)	220 (56)		1,573 (54.7)		0.558
Winter, *n* (%)	241 (62)		1,567 (55)		0.008
Age (years)	43.0	16.1	39.8	14.9	<0.001
Weight (kg)	75.6	16.4	73.1	15.3	0.002
Height (cm)	172	10	173	10	0.763
Bmi (kg/m^2^)	25.2	4.3	24.4	3.9	<0.001
SpO_2_ (%)	73.8	6.9	88.5	4.5	<0.001
Heart rate (bpm)	88.5	15.0	86.0	15.9	0.003
Systolic BP (mmHg)	125	18	124	18	0.497
Diastolic BP (mmHg)	74	8	73	7.4	0.323

## Discussion

The main findings of this observational study were that, under the conditions of our study (i.e., high altitude reached by cableway with data collection inside the station shortly upon arrival), SpO_2_ was associated with sex, age, BMI, HR, and season but not with BP. Three hundred ninety-one out of 3,267 subjects (12%) had an SpO_2_ ≤80% and were more likely to be older, have a BMI over 25.2 ± 4.3 kg/m^2^ and have ascended to Punta Helbronner during winter. Notably, none of the subjects who participated in the study reported symptoms associated with acute high-altitude exposure needing medical attention.

This is the first analysis of the effects of rapid high-altitude exposure in a large, unselected population. We were able to collect data of 1,808 subjects during the winter season of the Skyway operation (January 1 to March 8) and 1,459 subjects during the summer season (July 6 to October 18).

**FIG. 2.**
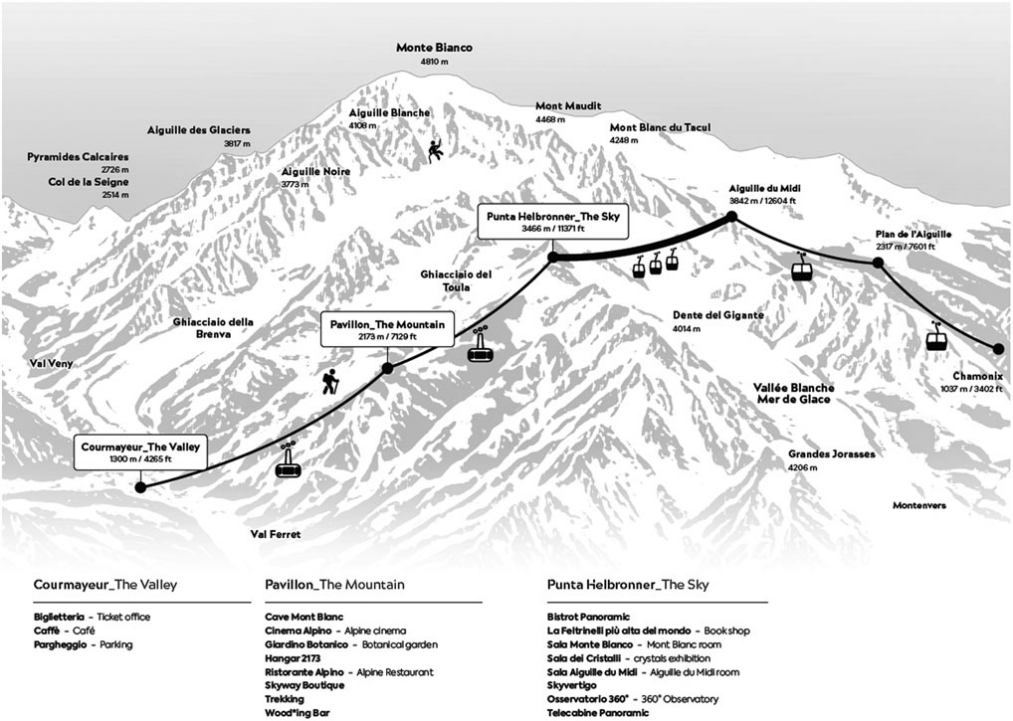
Skyway Monte Bianco and Trans Mont Blanc cableway system. Courtesy of Skyway Monte Bianco.

**FIG. 3.**
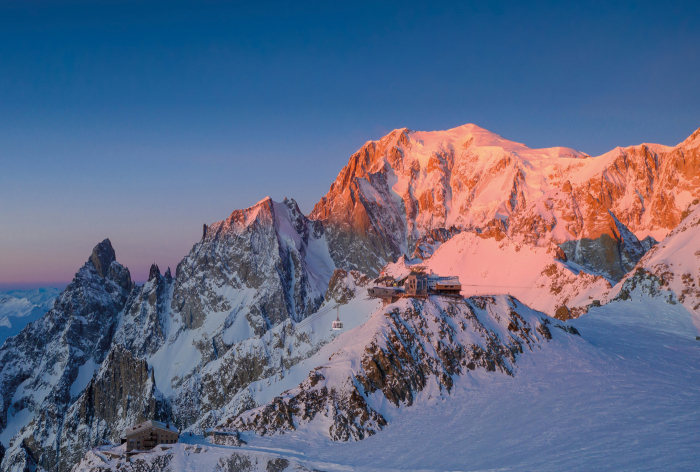
Panoramic view of Mont Blanc, Punta Helbronner Skyway station and Torino shelter. Courtesy of Skyway Monte Bianco.

Punta Helbronner is the third of the six stations of the Trans Mont Blanc cableway, which from Entreves (Courmayeur) reaches Pavillon station (2,173 m), Punta Helbronner and then crosses over the Mer de Glace glacier arriving first at the Aiguille du Midi (3,842 m), at Plan de l'Aiguille (2,317 m), and then descending to Chamonix (1,035 m) (Fig. 2). Punta Helbronner is a warm, quiet location suitable for measuring physiological parameters without disturbance. It features a bookshop, a nice cafeteria, a permanent crystal exhibition, and a wide circular terrace with 360° view and windows, allowing even from inside the station a breathtaking view of the Mont Blanc chain (Fig. 3).

The average transportation time from Courmayeur to Punta Helbronner is particularly short (a difference in altitude of 2,166 m in about 20 minutes), and measurements were obtained shortly upon arrival at Punta Helbronner. Moreover, the duration of stay in the high-altitude station was generally short, since overnight stay was not allowed.

The cohort we assessed included volunteers, and it represents a sample of subjects who normally reach high altitude for tourism. The great majority of subjects arriving at Punta Helbronner in wintertime just look outside, enjoy a quick lunch, or spend some time in the library and then go back to Courmayeur by cableway. Of note, the other cableway linking to France (the Trans-Mont Blanc cableway) is closed during the winter season. Moreover, both off-piste ski descent and winter excursions on Mont Blanc are only possible with professional guide assistance and both imply relevant mountain expertise and fitness. The population who reaches Punta Helbronner during the summer season is more or less of the same type as in the winter season, although a few more mountaineers are likely present, since Punta Helbronner is the starting point of several high-altitude summer excursions.

Regardless of these seasonal differences, individuals ascending in summer showed on average the same BMI of individuals ascending in winter, so that relevant differences in participants' fitness are unlikely. In both seasons, reaching Punta Helbronner for a short visit is safe and, indeed, no major health-related events were reported. As a matter of fact, subjects at higher risk, which we arbitrarily defined as those with SpO_2_ ≤80%, were older, more frequently males, and obese, and they more often reached Punta Helbronner during the winter season. The value of 80% was chosen as it was the median saturation recorded at Capanna Margherita, 4,554 m, that is, over 1,000 m above Punta Helbronner (Agostoni et al., 2010).

Of note, our data were collected shortly upon arrival at high altitude, so that we cannot speculate on what happens with a more prolonged exposure, considering that acute mountain sickness is usually reported after 12–24 hours of high-altitude exposure (Agostoni et al., 2010). Indeed, our study conditions fully respect the recommendations given by expert mountaineers, that is, climb high, sleep low (Agostoni et al., 2020). It should also be noted that data at sea level could not be collected due to logistic factors and to our study conditions. Therefore, we cannot determine whether our study results are only related to absolute high-altitude-induced SpO_2_ changes or also to the presence of a reduced SpO_2_ even at sea level.

Our study investigates the cardiovascular responses to an effortless acute exposure to high altitude. While differences in SpO_2_ changes with high-altitude exposure are expected between older and younger individuals as well as between overweight and lean subjects (Guo et al., 2014; Hsu et al., 2015; Richalet et al., 2020), sex- and season-related differences need some interpretation.

Female individuals showed a higher SpO_2_ even after correction for confounding factors, such as age, BMI, and season. It is recognized that the sex-related differences in SpO_2_ we observed are small and of uncertain significance. Regardless, a few speculations based on previous published and present data on the effects of high altitude on female cardiorespiratory physiology are possible. Indeed, it is known, however, that women appear to be somehow protected against the effects of high-altitude exposure, as shown by their lower sleep abnormality rates both with acute and prolonged high-altitude exposure (Lombardi et al., 2013; Burtscher et al., 2019; Richalet et al., 2020). Our findings are in line with these studies.

Several possible explanations have been suggested for the better gas exchange at high altitude in females, including differences in chemoreflex response, both peripheral and central, in respiratory center activities, in cerebral blood flow and muscle CO_2_ production at rest (White et al., 1982; Lahiri et al., 1983; Bradley et al., 1986; Saaresranta and Polo, 2002; Duffin, 2005; Lombardi et al., 2013; Caravita et al., 2015). Regardless of the underlying mechanisms, the present report adds some new information on this topic, showing for the first time that sex-related respiratory differences appear immediately after reaching high altitude, suggesting that chemoreflex mechanisms may be among the factors responsible for the reported sex differences in SpO_2_ (Lombardi et al., 2013; Caravita et al., 2015). The effect of season is also of interest although already well known. Differences in ambient temperature are possible but likely minor, since all data were collected inside the cableway station. Other possible factors responsible for our findings may be air humidity and barometric pressure, both more likely to be lower during the winter season (West et al., 1983).

Most importantly, summer data were obtained while wearing a facial mask, which clearly implies an extra work of breathing (Lassing et al., 2020; Hopkins et al., 2021; Mapelli et al., 2021). Nevertheless, recently published data show that even in patients with lung disease, SpO_2_ during a six-minute walking test is not worse with a mask (Just et al., 2021). It is possible, although yet unproven that season-related differences are affected by the wearing of facial masks, being actually greater than those reported by this study. The effects of face masks at high altitude need to be assessed by a dedicated trial. Similarly, we reported a correlation between high BMI and low SpO_2_ at high altitude. However, we do not know whether this is due to altered lung mechanics, altered control of breathing, or basilar atelectasis in obese subjects, and dedicated studies are needed on this topic.

Finally, we report a correlation of age with low SpO_2_ at high altitude but did not analyze any cause–effect relationship (Lhuissier et al., 2012). In brief, our study merely established some associations between acute high-altitude exposure and some cardiorespiratory variables, but it does not provide any sense of causality.

This is a pilot study, although obtained on a sizable cohort of subjects reaching high altitude for tourism in a very fast way (20 minutes). Accordingly, our results represent unique experimental observations, and data may be different from those obtained when high altitude is reached either by car or by hike (Agostoni et al., 2011, 2013; Caravita et al., 2014; Goodman, 2015). Moreover, this is the first of a series of further planned reports. Indeed, the permanence of a high-altitude automated Keito K9 biometric multiparametric recording system will allow other studies beyond this pilot analysis.

The effects of cigarette smoke and of antihypertensive treatment (some drugs such as β-blockers or AT1 receptor blockers have a direct action on chemoreflex response) (Agostoni et al., 2006; Marcus et al., 2010; Contini et al., 2013), the effects of season without the confounding effect of face masks, and the effect of face masks by themselves are clearly relevant information which will be collected in the future. Moreover, as soon as the present pandemic allows it, we will install a second Keito K9 biometric multiparametric recording system at Entreves (Courmayeur), where the Skyway departure station is located, which will allow us to assess the difference in cardiorespiratory variables caused by acute high-altitude exposure, having values recorded at a lower level as reference.

A few study limitations should be underlined. First, this is merely an observational study with no analysis of the reasons behind our findings. Second, we evaluated normal subjects at rest just upon arrival at high altitude. Accordingly, our data cannot be extrapolated in different settings such as prolonged permanence at high altitude, subjects with diseases or exposed to cold or bad weather, or subjects ascending to high altitude on foot or exercising. Furthermore, day-by-day barometric pressure data are not available. Finally, since ours is a completely automated station, it is not possible to exclude that the enrolled subjects spoke or changed their breathing during measurements, thus altering the results.

In conclusion, high BMI, older age, and male sex are strong predictors of significant blood oxygen desaturation following acute and fast exposure to high altitude, especially if the exposure occurs during the winter.

## Data Sharing

Raw data will be accessible upon request at www.zenodo.org
